# Detecting Overlapping Protein Complexes by Rough-Fuzzy Clustering in Protein-Protein Interaction Networks

**DOI:** 10.1371/journal.pone.0091856

**Published:** 2014-03-18

**Authors:** Hao Wu, Lin Gao, Jihua Dong, Xiaofei Yang

**Affiliations:** 1 School of Computer Science and Technology, Xidian University, Xi'an, Shaanxi, China; 2 Foreign Language Department, Northwest A&F University, Yangling, Shaanxi, China; Humboldt-Universität zu Berlin, Germany

## Abstract

In this paper, we present a novel rough-fuzzy clustering (RFC) method to detect overlapping protein complexes in protein-protein interaction (PPI) networks. RFC focuses on fuzzy relation model rather than graph model by integrating fuzzy sets and rough sets, employs the upper and lower approximations of rough sets to deal with overlapping complexes, and calculates the number of complexes automatically. Fuzzy relation between proteins is established and then transformed into fuzzy equivalence relation. Non-overlapping complexes correspond to equivalence classes satisfying certain equivalence relation. To obtain overlapping complexes, we calculate the similarity between one protein and each complex, and then determine whether the protein belongs to one or multiple complexes by computing the ratio of each similarity to maximum similarity. To validate RFC quantitatively, we test it in Gavin, Collins, Krogan and BioGRID datasets. Experiment results show that there is a good correspondence to reference complexes in MIPS and SGD databases. Then we compare RFC with several previous methods, including ClusterONE, CMC, MCL, GCE, OSLOM and CFinder. Results show the precision, sensitivity and separation are 32.4%, 42.9% and 81.9% higher than mean of the five methods in four weighted networks, and are 0.5%, 11.2% and 66.1% higher than mean of the six methods in five unweighted networks. Our method RFC works well for protein complexes detection and provides a new insight of network division, and it can also be applied to identify overlapping community structure in social networks and LFR benchmark networks.

## Introduction

In the past several years, large-scale proteomics experiments have produced many PPI data sets from different organisms [1]. These data sets are generally represented as undirected weighted or unweighted networks with proteins as a set of nodes and interactions as a set of edges. Edge weight estimates the reliability of such interaction. Protein-protein interactions play significant roles in cell's structural components and the process ranging from transcription, splicing site and translation to cell cycle control [2]. It is essential to extract overlapping protein complexes or regulatory pathways from PPI networks to investigate disease-related gene and drug target.

Densely connected regions in a graph can be identified by some unsupervised clustering method. However, many clustering methods are not ideal for PPI networks [1]. Some proteins may have multiple functions, hence the corresponding proteins could belong to more than one complex. Recently, a lot of clustering algorithms have been proposed to detect overlapping protein complexes in PPI networks [1], [3], [4], [5], [6], [7]. Each of them has limitations: some algorithms only work in unweighted networks, and can be applied to weighted data sets only after binarizing them by deleting edges whose weights are below a given threshold, while others need to assign the number of complexes firstly [8], [9]. Although the notion of the overlapping protein complexes is easy to understand, constructing an effective algorithm for overlapping protein complexes is highly non-trivial for two reasons: firstly, the number of protein complexes is unknown for a given PPI network; secondly, a protein complex should contain many reliable interactions within its subunit, and it should be well-separated from the rest of the PPI networks [1].

Fuzzy sets and rough sets have been widely applied to many fields, such as fuzzy clustering [10], [11], rough k-means clustering [9], [12], [13], [14], [15], fuzzy c-means clustering [16], [17], rough-fuzzy c-means clustering [18], [19], [20] and dynamic rough clustering [21], [22]. One of the most remarkable attempts to clustering problems may be c-means clustering and its derivatives. However, those algorithms are mainly applied to two dimensional microarray gene data, image data and forest cover rather than three dimensional network data, and mainly adapt rough set and fuzzy set theory to c-means clustering [18]. Those algorithms have the following weaknesses, firstly, the number of clusters *c* is an input parameter, and an inappropriate choice of *c* may yield poor results. In most cases, it is difficult to assess the numbers of clusters (*c value*) in original datasets. Thus, diagnostic checks have to be performed on and on to determine the number of clusters in the data set when performing *c*-means. Secondly, the choice of the initial cluster centers has a great impact on the clustering results; once the initial value selected is not good, it could not draw effective clustering results. Thirdly, the algorithm requires constant adjustment for sample classification and constantly calculating the adjusted new cluster centers, so when the data is very large, the algorithm time complexity will increase.

In order to solve the three dimensional datasets clustering problems in PPI networks and the weaknesses of c-means clustering, we present a novel method based on rough-fuzzy clustering (RFC) to detect overlapping protein complexes in PPI networks. RFC integrates the merits of fuzzy sets and rough sets, focuses on fuzzy relation model rather than graph model. RFC utilizes fuzzy set to create fuzzy relation between nodes and transform the fuzzy relation into fuzzy equivalence relation, and then create equivalence classes which correspond to non-overlapping protein complexes. The upper and lower approximations of rough sets are used to decide whether one protein belongs to one or more complexes, so we obtain overlapping complexes. RFC can automatically obtain the number of clustering by the number of equivalence classes, removing the limitation of selecting the initial clustering number. RFC also has advantage in datasets with large number of prototypes.

To test RFC's performance, we apply it to identify overlapping and non-overlapping community structure in artificial synthetic networks and social networks. To evaluate RFC quantitatively, we apply it to detect overlapping protein complexes in four weighted yeast data sets [23], [24], [25] and five unweighted yeast data sets [23], [24], [25], [26], and then we execute six other popular clustering methods (ClusterONE [1], CMC [27], MCL [28], GCE [29], OSLOM [30] and CFinder [3]) in the same data sets. Predicted complexes derived by the seven methods are separately compared with reference complexes from the Munich Information Centre for Protein Sequence (MIPS) [31] and the Saccharomyces Genome Database (SGD) [32]. Finally, results derived by the seven methods are compared with some evaluation criteria to assess RFC.

## Materials and Methods

### The definitions of rough-fuzzy clustering

Prior to providing a detailed description of our algorithm, we introduce some terminologies widely used in the forthcoming sections. Let 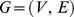 be an undirected graph, where *V* is a set of nodes, and *E* is a set of edges.

**Definition 1.** Let *N*(*u*) be the neighbors of node *u*. *Sim*(*u*, *v*), similarity for node pair *u* and *v*, is 1 if *u* = *v*; else 
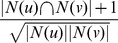
 if 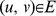; 0 otherwise.

Here, we define similarity between nodes based on their shared neighbors, if *u* and *v* are not directly neighbors, 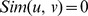; if *u* and *v* are directly neighbors, the more shared neighbors of *u* and *v*, the larger value of 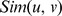; if *u* and *v* are the same node, 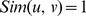, that is, 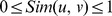. If two nodes have similar topological structure, they may share similar functions [11]. Similarity in network topological structure decides the degree of similarity between a pair of nodes.

**Definition 2.** Let *V* be a nonempty set, and *R* be an equivalence relation. For each 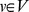, the equivalence class of object *v* for *R* is defined as follows [12]:
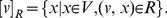
(1)


**Figure 1:**
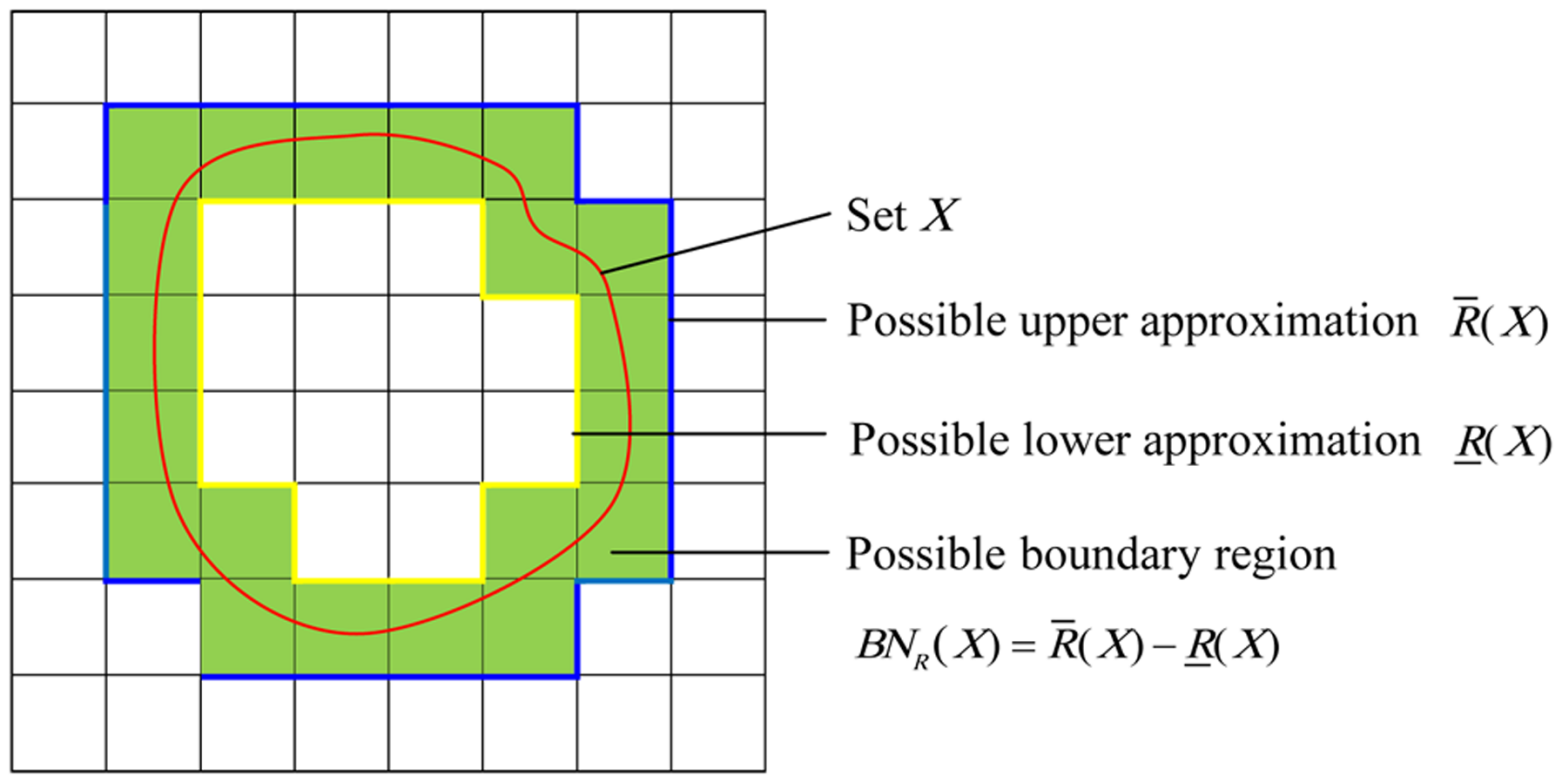
The relationship among Set *X* and its possible lower approximation, upper approximation and boundary region for equivalence relation *R*. In the figure, we provide the relationship among set *X*, lower approximation 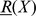, upper approximation 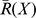 and boundary region 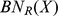. The internal region of the red curve represents set *X*, the internal region of the yellow line represents lower approximation 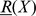, the green region represents boundary region 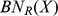, the internal region of the blue line represents upper approximation 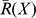, and the whole region represents universal set.

**Definition 3.** For set 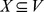, the upper and lower approximations of *X* for *R* are defined as follows, respectively [12]:
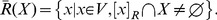
(2)

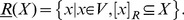
(3)Here, 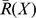 is the upper approximation of *X* for equivalence relation 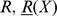 is the lower approximation of *X* for equivalence relation *R.* Obviously,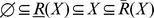. 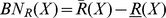 is called as boundary region of *X* for equivalence relation *R*, and their relationship is shown in Figure 1.

Let *u* be an object of set 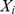. It is obvious in Figure 1 that the upper and lower approximations of 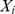 are only a few subsets of *V*. The family of the *k* upper and lower approximations of the 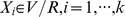 necessarily meet the following basic rough set properties [12]:

Property 1: An object *u* can be a part of at most one lower approximation.

Property 2: 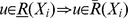.

Property 3: *u* is not a part of any lower approximation 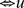 belongs to two or more boundary regions.

The next step is how to determine whether an object belongs to boundary region or lower approximation of a set. For each object *u*, let 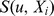 be similarity between *u* and any set 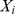. The definition of 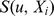 is as follows:

**Definition 4.** Similarity between node *u* and set *X_i_* is
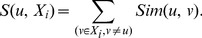
(4)Here, 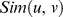 is obtained by Definition 1. The ratio 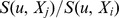 is used to decide the assignment of *u* as follows [12], [13]:

1. If 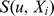 is the maximum for 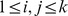 and 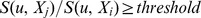 (*k* denotes the number of sets referring to the number of equivalence classes), 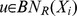 and 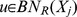. Furthermore, *u* is not a part of any lower approximation. This criterion ensures that Property 3 is satisfied.
2. Otherwise, 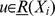 such that 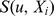 is the maximum for 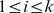. In addition, by Property 2, 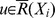. This criterion also satisfies Property 1.

### The rough-fuzzy clustering method

**Figure 2:**
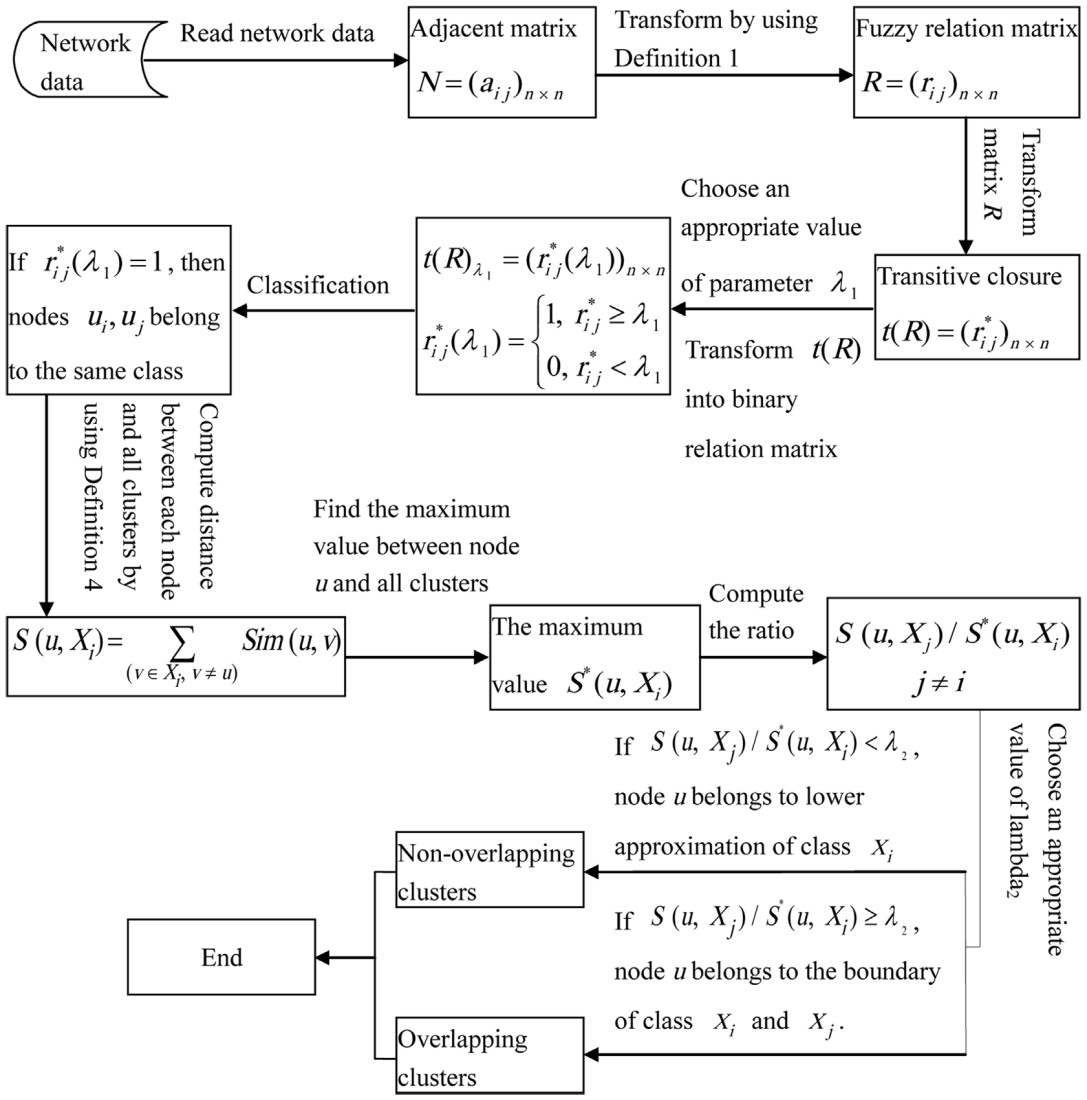
RFC algorithm flowchart. In the figure, we briefly give RFC algorithm flowchart to describe the operational process of the algorithm.

The RFC consists of the following major steps, as shown in Figure 2.

**Figure 3:**
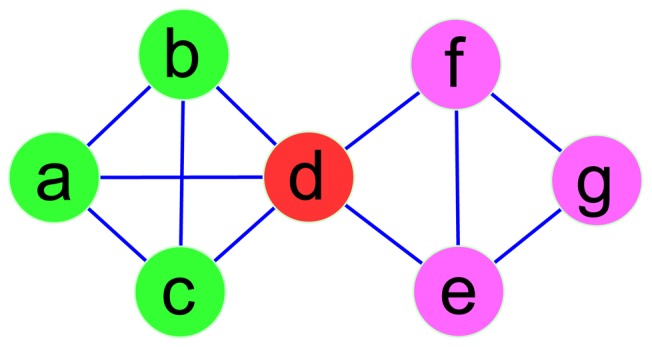
Artificial synthetic graph for illustrating the process of the rough-fuzzy clustering method. In the figure, the network is made of two communities and node *d* is overlapping node.

1. The graph (Figure 3) can be represented by an adjacency matrix *N*, and then transform the adjacency matrix *N* into the fuzzy matrix *R* by calculating the similarities between any two nodes (Definition 1). Obviously, *R* is reflexive and symmetric.
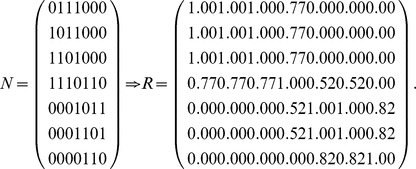

2. Transform the fuzzy matrix *R* into the fuzzy equivalence relation *t*(*R*) by transitive closure [33].
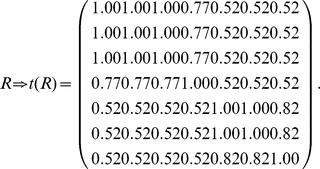

3. Choose a threshold 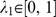 and transform *t*(*R*) as a Boolean equivalence relation 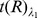. Let 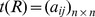 and 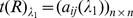. Here 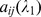 is 1 if 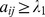, 0 otherwise. Therefore, different *λ*_1_ corresponds to different equivalence relations and equivalence classes as follows:
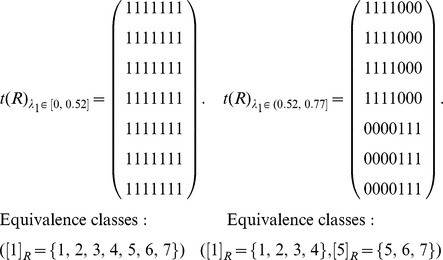


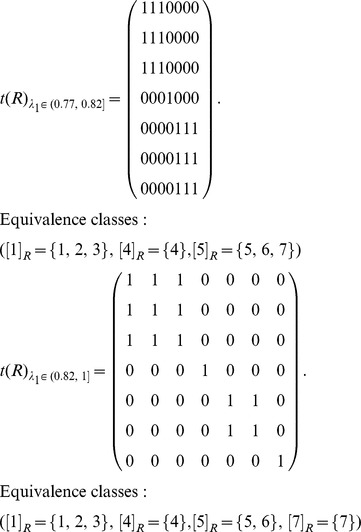

4. According to different *λ*_1_, *S*(*u*, *X_i_*) is computed by Definition 4. Here, each row represents a node, and each column represents an equivalence class which has been obtained in step (3). In the formula 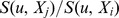, 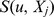 represents the similarity of node *u* and class *X_j_*, and *S*(*u*, *X_i_*) represents the maximum of similarities between node *u* and each class.
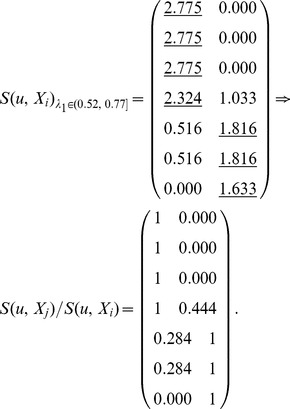
Here, 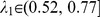, and these objects are classified into two equivalence classes: 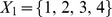, 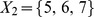. If 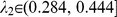, 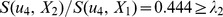. Therefore, *u*_4_ belongs to the boundary region of *X*_1_ and *X*_2_. In this case, non-overlapping sets, 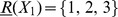 and 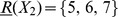, and overlapping sets 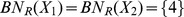 are obtained.
  The underlined numbers represent the maximum of similarity between each object and each class.
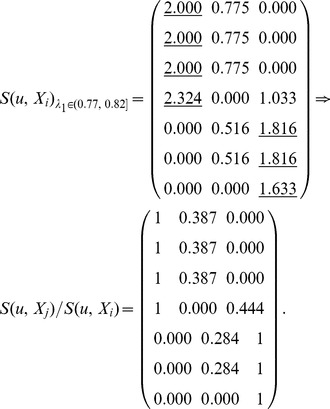
Here, 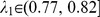, and these objects are classified into three equivalence classes: 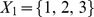, 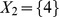, 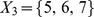. If 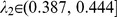, 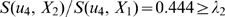. Therefore, *u*_4_ belongs to the boundary region of *X*_1_ and *X*_3_, 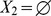. In this case, non-overlapping sets, 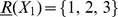 and 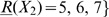, and overlapping sets 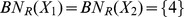 are obtained.
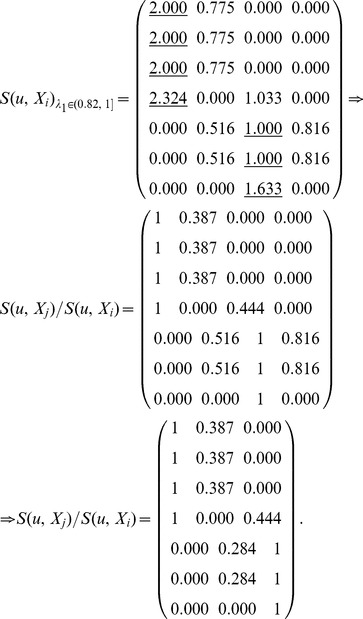
Here, 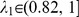, and these objects are classified into four equivalence classes: 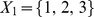, 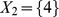, 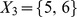, 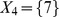. If 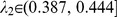
and *i* = 1, 2 and 4, 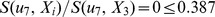. Therefore, *u*_7_ belongs to the lower approximation of 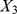 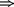 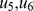 and 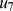 belong to the same equivalence class *X*_3_. If 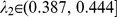, 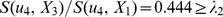. Therefore, *u*_4_ belongs to the boundary region of *X*_1_ and *X*_3_, 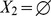. In this case, non-overlapping sets, 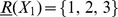 and 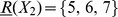, and overlapping sets 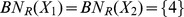 are obtained.
5. Merge the sets with overlapping degree to a very high extent in comparison with their sizes [1]. We evaluate the extent of overlapping between each pair of sets by formula 10 and merge the two sets whose overlapping score is above a specific threshold. Let merging threshold be 0.64, because it shows that the intersection is at least 80% of the size of the set if the two sets are equal in size.

We have discussed the details of RFC. The choice scale of *λ* is relatively larger and more flexible than fuzzy clustering, and the clustering results are relatively stable for different *λ*. In the following section, RFC will be applied in artificial synthetic networks, social networks and PPI networks.

### Parameter settings

In the algorithm, threshold *λ*_1_ is used to divide networks to get non-overlapping modules. The *λ*_1_ is closely related to the size of similarities of between nodes in all kinds of networks. Based on the analysis of the algorithm and a large number of experiments, we obtain *λ*_1_ according to the following formula:
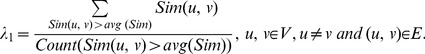
(5)


Here, 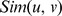 obtained by Definition 1 represents the similarity between nodes, 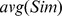 represents the mean of similarities of all pairs of nodes, and 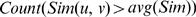 represents the number of the values that are greater than mean 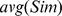.

Threshold *λ*_2_ is applied to determine whether one node belongs to one or multiple modules. In this article, it is set into an adjustable value. Based on a large number of experiments, it is a good choice to set 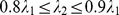.

### Evaluation criteria

Different criteria proposed by earlier studies are applied to evaluate RFC. The criteria are defined to assess the similarity between predicted modules and reference modules. The first measure is Normalized Mutual Information (NMI), which is an information theory based on quantifying the closeness of two groups of sets which has been widely used in clustering algorithms and machine learning [30], [34], [35], [36]. It is defined as:
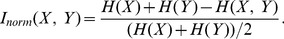
(6)


Here, 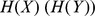 is the entropy of the random variable *X*(*Y*), whereas 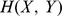 is the joint entropy.
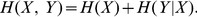
(7)

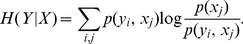
(8)

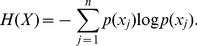
(9)


Here, for a random variable *X* with *n* outcomes 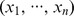, 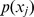 is the probability mass function of outcome 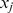, and 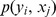 is the probability that 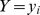 and 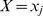.

The Second measure is the overlapping score between predicted and reference complexes, which is shown as follows [37]:
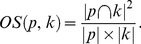
(10)Here, 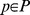 is a predicted complex and 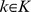 a reference complex. *P* is the set of predicted complexes and *K* is the set of reference complexes.

After defining overlapping score 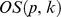 between predicted complex and reference complex, precision, recall and F1 measure are defined as follows [37]:
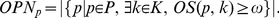
(11)

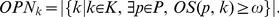
(12)

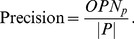
(13)

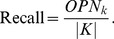
(14)

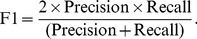
(15)Here, 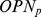 is the number of predicted complexes as 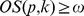 and 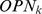 is the number of reference complexes as 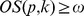. The overlapping threshold *ω* = 0.25 is chosen, because it shows that the intersection is at least half of the complex size if the two complexes are equal in size [1]. Precision is the fraction of the predicted complexes that match known complexes. Recall represents the fraction of known complexes that match predicted complexes. F1 measure gives a reasonable combination of both precision and recall.

Giving the known complexes as reference classification, we take sensitivity as the score of members of the *ith* known complex which are found in the *jth* predicted complex. Clustering-wise sensitivity (*Sn*) is defined as follows [1], [37]:
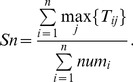
(16)Here, *n* is the number of known complexes. 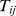 is the number of common proteins between the *ith* known complex and the *jth* predicted complex, and 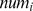 is the number of proteins belonging to the *ith* known complex.

The positive predictive value (*PPV*) is the fraction of members of the *jth* predicted complex which belongs to the *ith* known complex. PPV is defined as follows [37]:
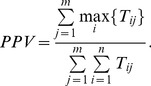
(17)Here, *m* is the number of predicted complexes, *n* is the number of known complexes.

The geometric accuracy (*Acc*) is the balance of both sensitivity and predictive value. It is obtained by calculating geometrical mean of *Sn* and *PPV* [37].
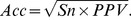
(18)


We employ separation to evaluate one-to-one correspondence between predicted complexes and known complexes. Separation of both the *ith* known complex and the *jth* predicted complex is shown as follows [1], [2], [37]:
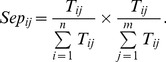
(19)

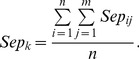
(20)

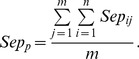
(21)

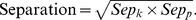
(22)Here, *n* is the number of known complexes. *m* is the number of predicted complexes. 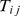 is the number of common proteins between the *ith* known complex and the *jth* predicted complex.

## Results

To validate RFC's feasibility, we apply it in artificial networks, social networks and protein interaction networks. In artificial networks, we compare its performance with those of the best algorithms currently available. The algorithms, GCE [21] and OSLOM [22] are selected for a fair comparison in LFR benchmark networks. To further verify the performance of our method, we apply RFC in Karate club network [38] and Dolphins network [39].

To evaluate RFC quantitatively, we apply it in four weighted and five unweighted large scale yeast PPI datasets (see Table 1), and compare predicted complexes with two reference complexes, MIPS [23] and SGD [24] (see Table 2). We also compare RFC results with those of six other popular methods, MCL [28], CFinder [3], ClusterONE [1], GCE [29], OSLOM [30] and CMC [5], [27] with an immediate purpose to test the performance of extracting overlapping complexes. The similarity in weighted networks is defined by weight of the edge, and the similarity in unweighted networks is calculated by definition 1.

**Table 1:** Initial datasets.

Unweighted networks	Weighted networks	Nodes numbers	Edges numbers	Density
Gavin [24]	Gavin [24]	1855	7669	4.134
Collins [23]	Collins [23]	1622	9074	5.594
Krogan_core [25]	Krogan_core [25]	2708	7123	2.630
Krogan_extended [25]	Krogan_extended [25]	3672	14317	3.899
BioGRID [26]	N/A	5640	59748	10.549

N/A represents that there is no weighted BioGRID network.

**Table 2:** Gold standard protein complexes.

General properties	MIPS [31]	SGD [32]
Protein numbers	1189	1279
Complex numbers	203	323
Overlapping proteins	401	296

### Artificial networks

The LFR [36] is a class of benchmark graphs which account for the heterogeneity in the distributions of node degrees and community sizes. It can be applied to overlapping communities, by assigning to each node the same number of neighbors in different communities. To simplify things, we suppose that each node belongs to the same number of communities [30]. Mixing parameter *u* as independent variable is the ratio of the number of external neighbors of a node by the total degree of the node [30]. Small values of *u* show well separated communities, whereas large values of *u* indicate high mixed to each other.

**Figure 4:**
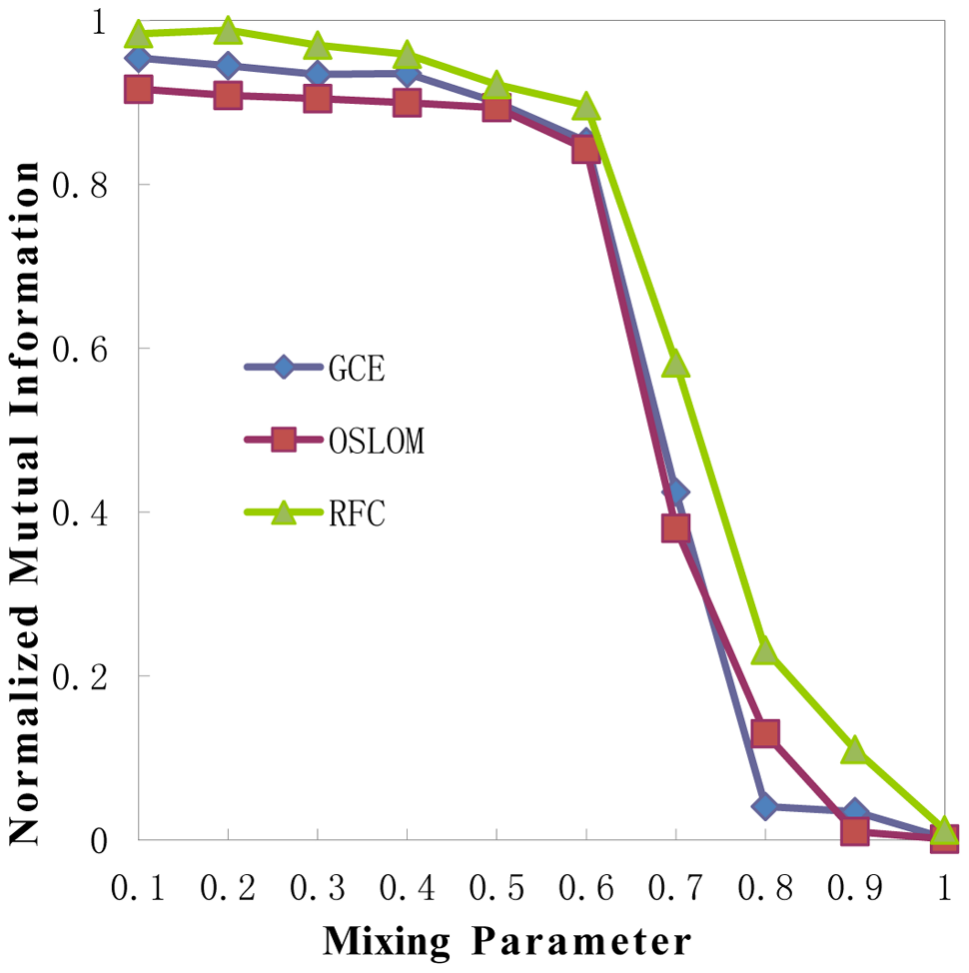
Results comparison of FRC, GCE and OSLOM in LFR benchmark graphs. The parameters of the graphs are: network size 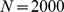, average degree 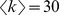, maximum degree 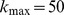, community size is in the range [20,50].

RFC is tested and compared with two recent methods, GCE [29], based on greedy clique expansion, and OSLOM [30], based on local optimization method. The two methods have good performances on LFR benchmark graphs with overlapping communities. The comparison of NMI's changes according to the mixture parameter *u* by three algorithms is presented in Figure 4

In all tests on LFR benchmark graphs, mixing parameter *u* varies from 0.1 to 0.9 with an interval 0.1 and each point is always 100 realizations, then mean of NMI is obtained as results. By increasing the value of *u*, communities become more and more fuzzy and it gets harder for any method to correctly detect the modules. We find that RFC performs competitively in comparison with GCE and OSLOM.

### Social networks

Although RFC performs well in artificial networks, we have to select two real-world networks for further evaluation.

### Karate club network

**Figure 5:**
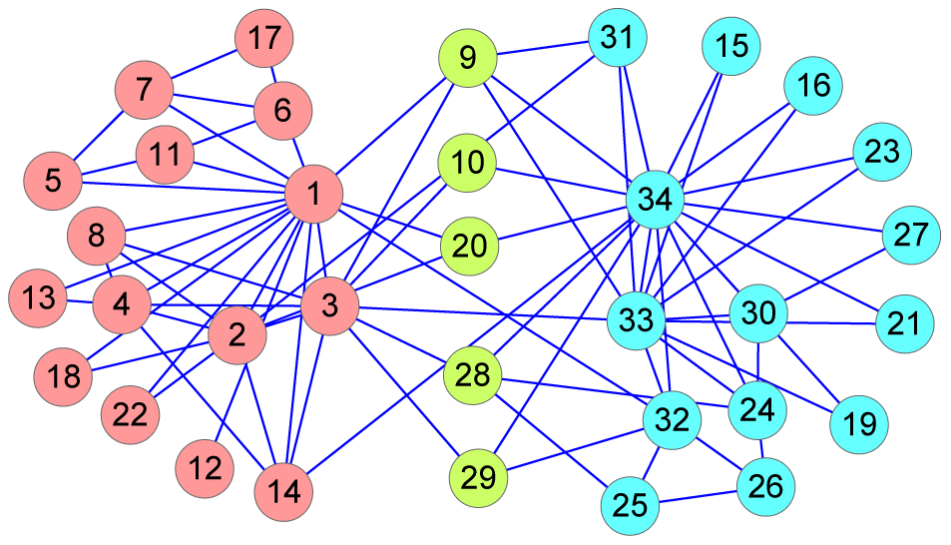
The RFC results for community structure in Zachary's karate club network. The divided result is shown for 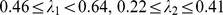. In the figure, dashed red nodes are fully assigned to the community which is centered at the club's instructor, dashed green nodes are completely assigned to the other community which is centered at the club's president, and dashed yellow nodes are shared between the two communities.

Zachary observed 34 members of a karate club at a US university in three years [38]. During the course of the time, node 1 (the club's instructor) and node 34 (the club's president) had some different ideas on the price of karate lessons. Ultimately the club was split into two organizations: one group was the supporters of the president and the other group was the supporters of the instructor. In fact, some individuals had friendship between the two groups, that is, these individuals may be overlapping nodes. Here we use an unweighted network version to test RFC and attempt to determine the factions involved in the split of the club. RFC performs well for detecting the two well-known communities which are centered at node 1 and node 34, respectively. The nodes 9, 10, 20, 28 and 29 are shared between the two groups. The communities coincide with overlapping nodes 9, 10, 20 observed by Sun et al. [10] with exception of nodes 28 and 29, which Sun et al. put with the community of the club's president. However, node 28 and node 29 have neighbors 3 and 34, respectively. Neighbor 34 is the club's president in one community, while neighbor 3 in the other community plays a pivotal role in its community. Therefore, it is reasonable that nodes 28 and 29 are overlapping. The detailed community structure of the network is shown in Figure 5.

### Dolphins network

**Figure 6:**
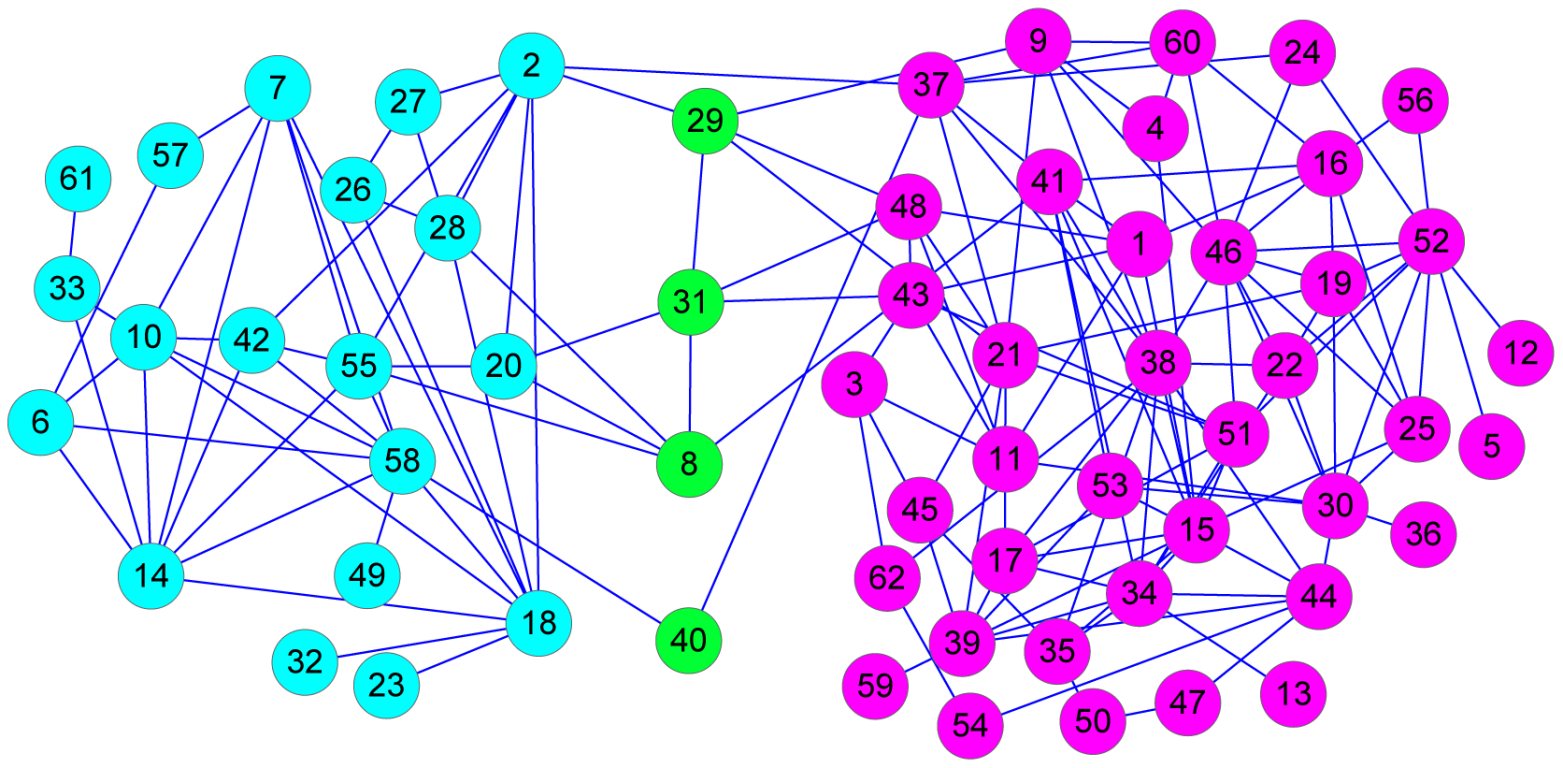
The RFC results for community structure in Lusseau's network of bottlenose dolphins.

The second example we discuss is the network studied by the biologist Lusseau [39], who divided a group of dolphins into two groups according to their age. There are 62 nodes and 159 edges in the network. RFC finds two communities with four overlapping nodes (8, 29, 31, 40), which can be seen in Figure 6. The partition of the two communities by RFC agrees with the separation observed by David Lusseau.

### PPI networks

**Figure 7:**
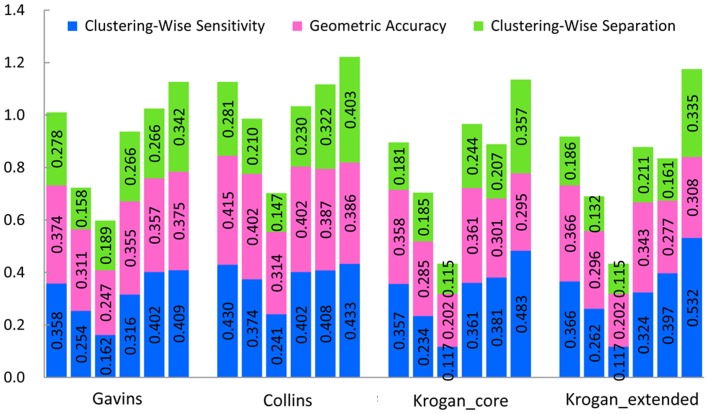
Results comparison of the six algorithms in four weighted datasets using MIPS gold standard. Columns correspond to the following algorithms, ClusterONE, CMC, CFinder, MCL, OSLOM and RFC from left to right in Gavins, Collins, Krogan_core and Krogan_extended weighted datasets, respectively, using MIPS gold standard. Various colors of the same column denote the individual components of the composite score of the algorithm (blue = the clustering-wise sensitivity, purple = geometric accuracy, green = the clustering-wise separation). The total height of each column is the value of the composite score for a special algorithm in a special dataset. Larger scores show the clustering result is better.

First, we test the six methods mentioned above in the weighted Gavin, Collins and Krogan datasets. Table 3 indicates the detailed benchmark results in Gavin dataset when the MIPS gold standard dataset is used as gold standard. The detailed benchmark results in Collins and Krogan datasets are provided in Table S1. Figure 7 gives results of a comparison of the six algorithms in the weighted Gavin, Collins, and Krogan datasets using MIPS gold standard. The results by RFC are compared with the ones by ClusterONE, CMC, MCL, OSLOM and CFinder. The precision, sensitivity and separation are 35.8%, 48.3% and 75.9% higher than mean of five other methods in the four weighted networks.

**Table 3:** Results of six protein complex detection algorithms in weighted Gavin dataset using MIPS gold standard.

Methods	#Complexes	Precision	F	Sensitivity	Accuracy	Sep_k_	Sep_p_	Separation
ClusterONE	196	0.536	0.526	0.358	0.374	0.274	0.283	0.278
CMC	341	0.416	0.522	0.254	0.311	0.205	0.122	0.158
CFinder	262	**0.591**	**0.666**	0.162	0.247	0.215	0.167	0.189
MCL	252	0.353	0.391	0.316	0.355	0.297	0.239	0.266
OSLOM	88	**0.625**	0.378	**0.402**	**0.357**	**0.175**	**0.404**	**0.266**
RFC	153	**0.575**	0.494	**0.409**	**0.375**	**0.297**	**0.394**	**0.342**

**Figure 8:**
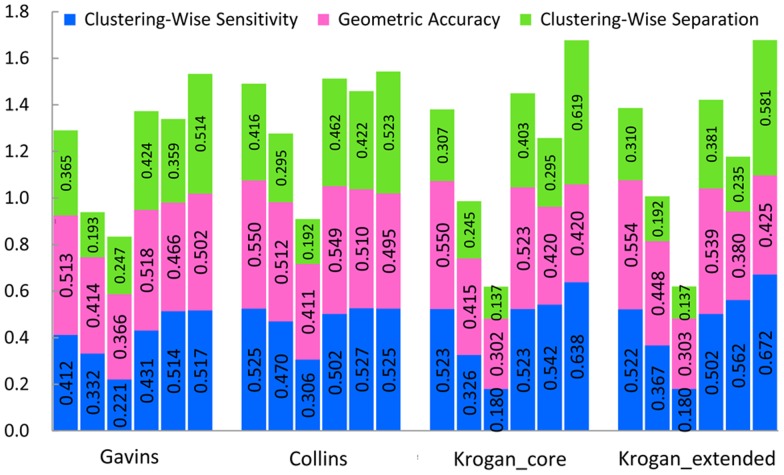
Results comparison of the six algorithms in four weighted datasets using SGD gold standard. Columns correspond to the following algorithms, ClusterONE, CMC, CFinder, MCL, OSLOM and RFC from left to right in Gavins, Collins, Krogan_core and Krogan_extended weighted datasets, respectively, using SGD gold standard. Various colors of the same column denote the individual components of the composite score of the algorithm (blue = the clustering-wise sensitivity, purple = geometric accuracy, green = the clustering-wise separation).

Table 4 indicates the detailed benchmark results in Gavin dataset when the SGD gold standard dataset is used as gold standard. The detailed benchmark results in Collins and Krogan datasets are provided in Table S2. Figure 8 gives results of a comparison of the six algorithms in the weighted Gavin, Collins, and Krogan datasets using SGD gold standard. The results by RFC are compared with the ones by ClusterONE, CMC, MCL, OSLOM and CFinder. The precision, sensitivity and separation are 29.7%, 38.9% and 85.9% higher than mean of five other methods in four weighted networks.

**Table 4:** Results of six protein complex detection algorithms in weighted Gavin dataset using SGD gold standard.

Methods	#Complexes	Precision	F	Sensitivity	Accuracy	Sep_k_	Sep_p_	Separation
ClusterONE	196	0.642	0.485	0.412	0.513	0.284	0.469	0.365
CMC	341	0.443	0.454	0.332	0.414	0.198	0.187	0.193
CFinder	262	**0.687**	**0.615**	0.221	0.366	0.222	0.274	0.247
MCL	252	0.488	0.428	0.431	**0.518**	**0.374**	0.480	0.424
OSLOM	88	**0.648**	0.277	**0.514**	0.466	0.187	**0.689**	**0.359**
RFC	153	**0.660**	0.424	**0.517**	0.502	0.353	**0.746**	**0.514**

**Figure 9:**
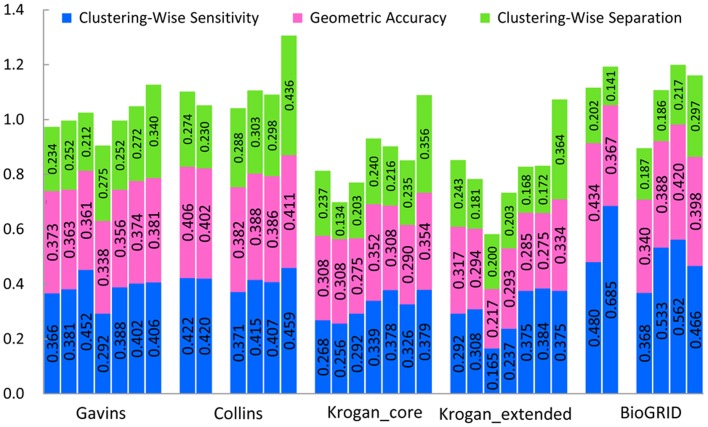
Results comparison of all the seven algorithms in five unweighted datasets using MIPS gold standard. Columns correspond to the various algorithms, ClusterONE, CMC, CFinder, MCL, OSLOM, GCE and RFC from left to right in Gavins, Collins, Krogan_core, Krogan_extended and BioGRID unweighted datasets, respectively, using MIPS gold standard. The two blank columns represent that CFinder algorithm does not give any result within 24 hours for Collins and BioGRID unweighted datasets.

Then we test all the seven methods mentioned above in the unweighted Gavin, Collins, Krogan, and BioGRID datasets. Table 5 indicates the detailed benchmark results in Gavin dataset when the MIPS gold standard dataset is used as gold standard. The detailed benchmark results in Collins, Krogan and Biogrid datasets are provided in Table S3. Figure 9 gives results of a comparison of all the seven algorithms in the unweighted Gavin, Collins, Krogan and Biogrid datasets using MIPS gold standard. RFC results are compared with ClusterONE, CMC, MCL, OSLOM, GCE and CFinder results. The precision, F1 measure, sensitivity, accuracy and separation are 0.1%, 16.1%, 10.5%, 9.6% and 60.5% higher than mean of six other methods in five unweighted networks.

**Table 5:** Results of seven protein complex detection algorithms in unweighted Gavin dataset using MIPS gold standard.

Methods	#Complexes	Precision	F	Sensitivity	Accuracy	Sep_k_	Sep_p_	Separation
ClusterONE	294	0.316	0.374	0.366	0.373	0.282	0.195	0.234
CMC	156	**0.532**	0.462	0.381	0.363	0.221	0.288	0.252
CFinder	184	0.359	0.341	**0.452**	0.361	0.202	0.223	0.212
MCL	228	0.364	0.385	0.292	0.338	0.291	0.259	0.275
OSLOM	105	**0.552**	**0.377**	**0.388**	**0.356**	**0.181**	**0.350**	**0.252**
GCE	117	**0.589**	**0.431**	**0.402**	**0.374**	**0.206**	**0.358**	**0.272**
RFC	187	**0.487**	**0.467**	**0.406**	**0.381**	**0.326**	**0.354**	**0.340**

**Figure 10:**
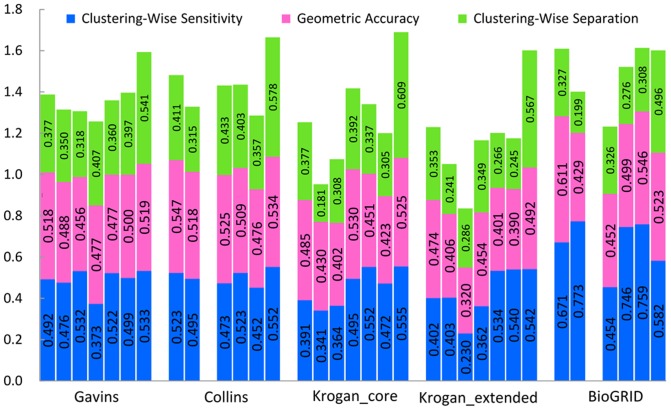
Results comparison of all the seven algorithms in five unweighted datasets using SGD gold standard. Columns correspond to the following algorithms, ClusterONE, CMC, CFinder, MCL, OSLOM, GCE and RFC from left to right in Gavins, Collins, Krogan_core, Krogan_extended and BioGRID unweighted datasets, respectively, using SGD gold standard. The two blank columns represent that CFinder algorithm does not give any result within 24 hours for Collins and BioGRID unweighted datasets.

Table 6 indicates the detailed benchmark results in Gavin dataset when the SGD gold standard dataset is used as gold standard. The detailed benchmark results in Collins, Krogan and Biogrid datasets are provided in Table S4. Figure 10 shows results of a comparison of all the seven algorithms in the unweighted Gavin, Collins, and Krogan datasets using SGD gold standard. RFC results are compared with ClusterONE, CMC, MCL, OSLOM, GCE and CFinder results. The precision, F1 measure, sensitivity, accuracy and separation are 2.7%, 26.6%, 11.8%, 10.1% and 69.8% higher than mean of six other methods in five unweighted networks.

**Table 6:** Results of seven protein complex detection algorithms in unweighted Gavin dataset using SGD gold standard.

Methods	#Complexes	Precision	F	Sensitivity	Accuracy	Sep_k_	Sep_p_	Separation
ClusterONE	294	0.395	0.376	0.492	0.518	0.360	0.395	0.377
CMC	156	0.583	0.380	0.476	0.488	0.243	0.503	0.350
CFinder	184	0.446	0.323	0.532	0.456	0.240	0.421	0.318
MCL	228	0.491	0.406	0.373	0.477	0.342	0.484	0.407
OSLOM	105	**0.562**	**0.276**	**0.522**	**0.477**	**0.205**	**0.632**	**0.360**
GCE	117	**0.666**	**0.354**	**0.499**	**0.500**	**0.239**	**0.661**	**0.397**
RFC	187	**0.626**	**0.459**	**0.533**	**0.519**	**0.412**	**0.711**	**0.541**

## Conclusion and Discussion

In this paper, we present a novel method based on rough-fuzzy clustering to detect overlapping and non-overlapping protein complexes in PPI networks. RFC is based on a fuzzy relation model which is transformed into equivalent classes to detect non-overlapping protein complexes. We further apply the upper approximation and lower approximation in rough sets to deal with each node in the network which belongs to one or multiple complexes. Ultimately, each complex corresponds to an overlapping protein complex.

RFC is tested in artificial networks, social networks and PPI networks and it is proved to provide a new insight into network division and to accurately recover communities in artificial networks. To determine whether these results are robust, we perform comparative benchmarks on a range of LFR graphs with overlapping communities, and find RFC performs competitively in comparison with GCE and OSLOM. To complete our evaluation, we test RFC and six other popular clustering algorithms in five unweighted PPI networks and four weighted PPI networks, and compare the results with MIPS and SGD gold standard datasets separately. We discover the three quality scores (accuracy, sensitivity and separation) obtained by RFC are obviously larger than those by six other methods.

Our results indicate that RFC outperforms six other popular algorithms in terms of matching more complexes between known complexes and predicted complexes with a higher accuracy, known complexes matching more predicted complexes with a higher sensitivity and providing a better one-to-one mapping with reference complexes with a higher separation. RFC results have a significant comprehensive advantage, especially in the Gavin and Collins datasets whose node numbers are close to the ones of the reference complexes. ClusterONE, OSLOM, GCE and MCL yield the closest score to RFC.

There exist several rough-fuzzy clustering algorithms in previous studies [8], [14], [17], [18], [40], such as rough c-means clustering (RCM) [13], [15], rough-fuzzy c-means clustering (RFCM) [8], [18] and rough-fuzzy possibilistic c-means clustering (RFPCM) [17]. These algorithms are mainly based on rough-fuzzy c-means clustering and its derivatives, and they are used to cluster co-expressed genes or functionally similar genes from microarray gene expression data sets. Recently, fuzzy-rough supervised gene clustering algorithm (FRSAC) has been proposed in [40] to detect groups of co-regulated genes whose expression is strongly associated with sample categories. The research objects of these clustering algorithms are two-dimensional gene expression data, that is, each row represents a gene and each column a sample. In those algorithms, the function of fuzzy sets is to handle overlapping partitions, and rough sets deal with uncertainty, vagueness, and incompleteness in class definition.

To our best knowledge, fuzzy clustering algorithm is firstly proposed in [11] to detect overlapping and non-overlapping community in social networks. In the algorithm, the choice of two thresholds is sensitive and it is difficult to choose accurate thresholds in large social networks and PPI networks. If the first threshold is not precise enough, some nodes supposed to belong to a community may not belong to any equivalence classes, so the nodes will not be allocated to the community. If the second threshold is not accurate enough, the overlapping nodes supposed to belong to two or multiple communities may not be allocated to the communities unless they have to be high correlated with the communities. Therefore, choosing the threshold values may cause some difficulties in large social networks and PPI networks and inaccuracy by excluding some edge nodes.

In order to solve the weaknesses, we propose a new algorithm RFC with different algorithms basis, clustering objects structure and the functions of rough set and fuzzy set. To be more specific, RFC algorithm is not based on c-means clustering, and the research objects of RFC are three-dimensional network data. In RFC, Fuzzy sets are used to create fuzzy equivalence relation and obtain clustering number automatically by calculating the number of equivalence classes. Rough sets are used to determine whether each node belongs to one or multiple complexes. The computing process of RFC indicates that the choice scale of the two thresholds in RFC is relatively larger and more flexible than fuzzy clustering algorithm [11]. It is also easier to detect the edge nodes for a community or a complex by introducing the upper and lower approximation in rough set than fuzzy clustering algorithm. The most significant advantage of RFC is that its separation is larger than the one in other algorithms, thus better evaluating one-to-one correspondence between predicted complexes and known complexes.

Protein complexes are key components to perform cellular functions associated with specific diseases [41], for example, overlapping proteins among multiple complexes tend to be drug targets [41]. In biological networks, some critical genes or motifs participate in multiple biological processes, implying the existence of overlapping modules. Studying the overlapping modules in networks is critical since it helps to confer the relationship between structure and function. In future work, we will focus on detecting human protein complexes to investigate disease related gene and drug target by RFC.

## Supporting Information

Table S1 **Results of six protein complex detection algorithms in weighted Collins, Krogan_core and Krogan_extended datasets using MIPS gold standard.**
(DOCX)

Table S2 **Results of six protein complex detection algorithms in weighted Collins, Krogan_core and Krogan_extended datasets using SGD gold standard.**
(DOCX)

Table S3 **Results of seven protein complex detection algorithms in unweighted Collins, Krogan_core, Krogan_extended and Biogrid datasets using MIPS gold standard.**
(DOCX)

Table S4 **Results of seven protein complex detection algorithms in unweighted Collins, Krogan_core, Krogan_extended and Biogrid datasets using SGD gold standard.**
(DOCX)
