# Supplementary material for: Detecting Overlapping Protein Complexes by Rough-Fuzzy Clustering in Protein-Protein Interaction Networks
Source: PLoS One. 2014 Mar 18;9(3):e91856. doi: 10.1371/journal.pone.0091856 (PMC3958373; doi:10.1371/journal.pone.0091856)
Supplement: Table S1 — Results of six protein complex detection algorithms in weighted Collins, Krogan_core and Krogan_extended datasets using MIPS gold standard. (DOCX) [file pone.0091856.s001.docx]

## Table S1: Results of six protein complex detection algorithms in three weighted PPI datasets using MIPS gold standard.

| Datasets | Methods | #Complexes | Precision | F | Sn | Acc | Sep_k_ | Sep_p_ | Sep |
| --- | --- | --- | --- | --- | --- | --- | --- | --- | --- |
| Collins | ClusterONE | 195 | 0.625 | 0.613 | 0.430 | **0.415** | 0.276 | 0.287 | 0.281 |
|  | CMC | 327 | 0.434 | 0.535 | 0.374 | 0.402 | 0.266 | 0.165 | 0.210 |
|  | CFinder | 361 | 0.468 | 0.599 | 0.241 | 0.314 | 0.196 | 0.110 | 0.147 |
|  | MCL | 180 | 0.628 | 0.590 | 0.402 | 0.402 | 0.282 | 0.318 | 0.230 |
|  | OSLOM | 99 | **0.909** | 0.596 | 0.408 | 0.387 | 0.225 | 0.461 | 0.322 |
|  | RFC | 108 | **1.000** | **0.695** | **0.433** | 0.386 | **0.294** | **0.553** | **0.403** |
| Krogan_  core | ClusterONE | 522 | 0.228 | 0.328 | 0.357 | 0.358 | 0.290 | 0.113 | 0.181 |
|  | CMC | 142 | **0.549** | 0.452 | 0.234 | 0.285 | 0.155 | 0.221 | 0.185 |
|  | CFinder | 374 | 0.417 | **0.540** | 0.117 | 0.202 | 0.157 | 0.085 | 0.115 |
|  | MCL | 366 | 0.232 | 0.298 | 0.361 | **0.361** | 0.327 | 0.182 | 0.244 |
|  | OSLOM | 58 | 0.138 | 0.061 | 0.381 | 0.301 | 0.110 | **0.385** | 0.207 |
|  | RFC | 122 | 0.361 | 0.271 | **0.483** | 0.295 | **0.473** | **0.270** | **0.357** |
| Krogan_  extended | ClusterONE | 530 | 0.270 | 0.390 | 0.366 | **0.366** | 0.300 | 0.115 | 0.186 |
|  | CMC | 368 | 0.288 | 0.371 | 0.262 | 0.296 | 0.177 | 0.098 | 0.132 |
|  | CFinder | 374 | **0.417** | **0.540** | 0.117 | 0.202 | 0.157 | 0.085 | 0.115 |
|  | MCL | 516 | 0.147 | 0.211 | 0.324 | 0.343 | 0.337 | 0.132 | 0.211 |
|  | OSLOM | 51 | 0.078 | 0.031 | 0.397 | 0.277 | 0.080 | **0.320** | 0.161 |
|  | RFC | 139 | **0.331** | 0.269 | **0.532** | 0.308 | **0.475** | **0.237** | **0.335** |
