# Supplementary material for: Detecting Overlapping Protein Complexes by Rough-Fuzzy Clustering in Protein-Protein Interaction Networks
Source: PLoS One. 2014 Mar 18;9(3):e91856. doi: 10.1371/journal.pone.0091856 (PMC3958373; doi:10.1371/journal.pone.0091856)
Supplement: Table S2 — Results of six protein complex detection algorithms in weighted Collins, Krogan_core and Krogan_extended datasets using SGD gold standard. (DOCX) [file pone.0091856.s002.docx]

## Table S2: Results of six protein complex detection algorithms in three weighted PPI datasets using SGD gold standard.

| Datasets | Methods | #Complexes | Precision | F | Sn | Acc | Sep_k_ | Sep_p_ | Sep |
| --- | --- | --- | --- | --- | --- | --- | --- | --- | --- |
| Collins | ClusterONE | 195 | 0.713 | 0.536 | 0.525 | **0.550** | 0.324 | 0.536 | 0.416 |
|  | CMC | 327 | 0.507 | 0.510 | 0.470 | 0.512 | 0.296 | 0.293 | 0.295 |
|  | CFinder | 361 | 0.540 | **0.570** | 0.306 | 0.411 | 0.203 | 0.181 | 0.192 |
|  | MCL | 180 | 0.783 | 0.560 | 0.502 | 0.549 | **0.345** | 0.619 | 0.462 |
|  | OSLOM | 99 | **0.969** | 0.455 | **0.527** | 0.510 | 0.234 | 0.762 | 0.422 |
|  | RFC | 108 | **0.972** | 0.487 | **0.525** | 0.495 | 0.302 | **0.904** | **0.523** |
| Krogan_  core | ClusterONE | 522 | 0.377 | 0.466 | 0.523 | **0.550** | 0.390 | 0.241 | 0.307 |
|  | CMC | 142 | **0.648** | 0.395 | 0.326 | 0.415 | 0.162 | 0.370 | 0.245 |
|  | CFinder | 374 | 0.521 | **0.559** | 0.180 | 0.302 | 0.147 | 0.127 | 0.137 |
|  | MCL | 366 | 0.338 | 0.360 | 0.523 | 0.523 | 0.429 | 0.378 | 0.403 |
|  | OSLOM | 58 | 0.396 | 0.121 | 0.542 | 0.420 | 0.125 | **0.697** | 0.295 |
|  | RFC | 122 | **0.546** | 0.297 | **0.638** | 0.420 | **0.650** | **0.590** | **0.619** |
| Krogan_  extended | ClusterONE | 530 | 0.384 | 0.478 | 0.522 | **0.554** | 0.397 | 0.242 | 0.310 |
|  | CMC | 368 | 0.329 | 0.350 | 0.367 | 0.448 | 0.205 | 0.180 | 0.192 |
|  | CFinder | 374 | **0.521** | **0.559** | 0.180 | 0.303 | 0.147 | 0.127 | 0.137 |
|  | MCL | 516 | 0.230 | 0.284 | 0.502 | 0.539 | 0.482 | 0.301 | 0.381 |
|  | OSLOM | 51 | 0.118 | 0.032 | 0.562 | 0.380 | 0.093 | **0.591** | 0.235 |
|  | RFC | 139 | **0.489** | 0.294 | **0.672** | 0.425 | **0.652** | **0.518** | **0.581** |
