# Supplementary material for: Detecting Overlapping Protein Complexes by Rough-Fuzzy Clustering in Protein-Protein Interaction Networks
Source: PLoS One. 2014 Mar 18;9(3):e91856. doi: 10.1371/journal.pone.0091856 (PMC3958373; doi:10.1371/journal.pone.0091856)
Supplement: Table S3 — Results of seven protein complex detection algorithms in unweighted Collins, Krogan_core, Krogan_extended and Biogrid datasets using MIPS gold standard. (DOCX) [file pone.0091856.s003.docx]

## Table S3: Results of seven protein complex detection algorithms in four unweighted PPI datasets using MIPS gold standard.

N/A represents that CFinder algorithm does not give any result within 24 hours for Collins and BioGRID datasets.

| Datasets | Methods | #Complexes | Precision | F | Sn | Acc | Sep_k_ | Sep_p_ | Sep |
| --- | --- | --- | --- | --- | --- | --- | --- | --- | --- |
| Collins | ClusterONE | 203 | 0.561 | 0.561 | 0.422 | 0.406 | 0.274 | 0.274 | 0.274 |
|  | CMC | 250 | 0.484 | 0.534 | 0.420 | 0.402 | 0.255 | 0.207 | 0.230 |
|  | CFinder | N/A | N/A | N/A | N/A | N/A | N/A | N/A | N/A |
|  | MCL | 183 | 0.568 | 0.538 | 0.371 | 0.382 | 0.274 | 0.304 | 0.288 |
|  | OSLOM | 110 | 0.836 | 0.587 | 0.415 | 0.388 | 0.224 | 0.412 | 0.303 |
|  | GCE | 75 | **0.980** | 0.553 | 0.407 | 0.386 | 0.181 | 0.490 | 0.298 |
|  | RFC | 152 | 0.743 | **0.637** | **0.459** | **0.411** | **0.379** | **0.502** | **0.436** |
| Krogan_  core | ClusterONE | 242 | 0.405 | **0.440** | 0.268 | 0.308 | 0.259 | 0.217 | 0.237 |
|  | CMC | 297 | 0.340 | 0.404 | 0.256 | 0.308 | 0.162 | 0.110 | 0.134 |
|  | CFinder | 115 | **0.487** | 0.352 | 0.292 | 0.275 | 0.153 | 0.270 | 0.203 |
|  | MCL | 373 | 0.214 | 0.278 | 0.339 | 0.352 | 0.325 | 0.177 | 0.240 |
|  | OSLOM | 86 | 0.384 | 0.228 | 0.378 | 0.308 | 0.141 | 0.332 | 0.216 |
|  | GCE | 68 | 0.485 | 0.243 | 0.326 | 0.290 | 0.136 | 0.407 | 0.235 |
|  | RFC | 304 | 0.267 | 0.320 | **0.379** | **0.354** | **0.445** | **0.286** | **0.356** |
| Krogan_  extended | ClusterONE | 239 | 0.397 | **0.430** | 0.292 | 0.317 | 0.264 | 0.224 | 0.243 |
|  | CMC | 105 | **0.504** | 0.344 | 0.308 | 0.294 | 0.130 | 0.252 | 0.181 |
|  | CFinder | 121 | 0.273 | 0.204 | 0.165 | 0.217 | 0.154 | 0.259 | 0.200 |
|  | MCL | 534 | 0.103 | 0.149 | 0.237 | 0.293 | 0.328 | 0.125 | 0.203 |
|  | OSLOM | 73 | 0.192 | 0.101 | 0.375 | 0.285 | 0.101 | 0.280 | 0.168 |
|  | GCE | 68 | 0.323 | 0.162 | **0.384** | 0.275 | 0.099 | 0.296 | 0.172 |
|  | RFC | 235 | 0.264 | 0.283 | **0.375** | **0.334** | **0.391** | **0.338** | **0.364** |
| BioGRID | ClusterONE | 473 | 0.279 | **0.390** | 0.480 | **0.434** | 0.308 | 0.132 | 0.202 |
|  | CMC | 114 | **0.439** | 0.315 | **0.685** | 0.367 | 0.105 | 0.188 | 0.141 |
|  | CFinder | N/A | N/A | N/A | N/A | N/A | N/A | N/A | N/A |
|  | MCL | 334 | 0.135 | 0.167 | 0.368 | 0.340 | 0.240 | 0.146 | 0.187 |
|  | OSLOM | 109 | 0.266 | 0.186 | 0.533 | 0.388 | 0.136 | 0.254 | 0.186 |
|  | GCE | 204 | 0.255 | 0.256 | 0.562 | **0.420** | 0.217 | 0.217 | 0.217 |
|  | RFC | 319 | 0.264 | **0.322** | 0.466 | **0.398** | **0.372** | **0.237** | **0.297** |
