# Supplementary material for: Detecting Overlapping Protein Complexes by Rough-Fuzzy Clustering in Protein-Protein Interaction Networks
Source: PLoS One. 2014 Mar 18;9(3):e91856. doi: 10.1371/journal.pone.0091856 (PMC3958373; doi:10.1371/journal.pone.0091856)
Supplement: Table S4 — Results of seven protein complex detection algorithms in unweighted Collins, Krogan_core, Krogan_extended and Biogrid datasets using SGD gold standard. (DOCX) [file pone.0091856.s004.docx]

## Table S4: Results of seven protein complex detection algorithms in four unweighted PPI datasets using SGD gold standard.

N/A represents that CFinder algorithm does not give any result within 24 hours for Collins and BioGRID datasets.

| Datasets | Methods | #Complexes | Precision | F | Sn | Acc | Sep_k_ | Sep_p_ | Sep |
| --- | --- | --- | --- | --- | --- | --- | --- | --- | --- |
| Collins | ClusterONE | 203 | 0.694 | **0.536** | 0.523 | **0.547** | 0.326 | 0.519 | 0.411 |
|  | CMC | 250 | 0.592 | 0.516 | 0.495 | 0.518 | 0.277 | 0.357 | 0.315 |
|  | CFinder | N/A | N/A | N/A | N/A | N/A | N/A | N/A | N/A |
|  | MCL | 183 | 0.721 | 0.521 | 0.473 | 0.525 | 0.326 | 0.576 | 0.433 |
|  | OSLOM | 110 | 0.900 | 0.457 | 0.523 | 0.509 | 0.235 | 0.690 | 0.403 |
|  | GCE | 75 | **0.982** | 0.377 | 0.452 | 0.476 | 0.172 | 0.742 | 0.357 |
|  | RFC | 152 | 0.835 | **0.534** | **0.552** | **0.534** | **0.397** | **0.839** | **0.578** |
| Krogan_  core | ClusterONE | 242 | 0.516 | **0.442** | 0.391 | 0.485 | 0.326 | 0.435 | 0.377 |
|  | CMC | 297 | 0.444 | 0.426 | 0.341 | 0.430 | 0.173 | 0.188 | 0.181 |
|  | CFinder | 115 | 0.669 | 0.351 | 0.364 | 0.402 | 0.184 | 0.517 | 0.308 |
|  | MCL | 373 | 0.321 | 0.344 | 0.495 | **0.530** | 0.421 | 0.365 | 0.392 |
|  | OSLOM | 86 | 0.581 | 0.244 | 0.552 | 0.451 | 0.174 | 0.653 | 0.337 |
|  | GCE | 68 | **0.750** | 0.261 | 0.472 | 0.423 | 0.140 | **0.665** | 0.305 |
|  | RFC | 304 | 0.418 | **0.405** | **0.555** | **0.525** | **0.603** | 0.616 | **0.609** |
| Krogan_  extended | ClusterONE | 239 | 0.506 | **0.431** | 0.402 | 0.474 | 0.304 | 0.410 | 0.353 |
|  | CMC | 105 | **0.667** | 0.327 | 0.403 | 0.406 | 0.138 | 0.423 | 0.241 |
|  | CFinder | 121 | 0.413 | 0.225 | 0.230 | 0.320 | 0.175 | 0.468 | 0.286 |
|  | MCL | 534 | 0.182 | 0.226 | 0.362 | 0.454 | 0.449 | 0.272 | 0.349 |
|  | OSLOM | 73 | 0.315 | 0.116 | 0.534 | 0.401 | 0.127 | 0.560 | 0.266 |
|  | GCE | 68 | 0.456 | 0.158 | 0.540 | 0.390 | 0.112 | 0.537 | 0.245 |
|  | RFC | 235 | 0.396 | **0.334** | **0.542** | **0.492** | **0.483** | **0.664** | **0.567** |
| BioGRID | ClusterONE | 473 | 0.410 | **0.487** | 0.671 | **0.611** | 0.396 | 0.270 | 0.327 |
|  | CMC | 114 | **0.509** | 0.265 | **0.773** | 0.429 | 0.118 | 0.335 | 0.199 |
|  | CFinder | N/A | N/A | N/A | N/A | N/A | N/A | N/A | N/A |
|  | MCL | 334 | 0.254 | 0.258 | 0.454 | 0.452 | 0.332 | 0.321 | 0.326 |
|  | OSLOM | 109 | 0.303 | 0.153 | 0.746 | 0.499 | 0.161 | 0.476 | 0.276 |
|  | GCE | 204 | 0.353 | 0.273 | 0.759 | **0.546** | 0.248 | 0.387 | 0.308 |
|  | RFC | 319 | **0.420** | **0.418** | 0.582 | **0.523** | **0.501** | **0.490** | **0.496** |
